# A global prediction model for sudden stops of capital flows using decision trees

**DOI:** 10.1371/journal.pone.0228387

**Published:** 2020-02-12

**Authors:** M. Belén Salas, David Alaminos, Manuel Angel Fernández, Francisco López-Valverde

**Affiliations:** 1 PhD Program in Economics and Business, Universidad de Málaga, Málaga, Spain; 2 Department of Finance and Accounting, Universidad de Málaga, Málaga, Spain; 3 PhD Program in Mechanical Engineering and Energy Efficiency, Universidad de Málaga, Málaga, Spain; 4 Department of Languages and Computer Science, Universidad de Málaga, Málaga, Spain; City University of Hong Kong, HONG KONG

## Abstract

Capital flows is an important aspect of the international monetary system because they provide great direct and indirect benefits, and at the same time, they carry risks of vulnerability for countries with an open economy. Numerous works have studied the behavior of these flows and have developed models to predict sudden stop events. However, the existing models have limitations and the literature demands more research on the subject given that the accuracy of the models is still poor, and they have only been developed for emerging countries. This paper presents a new prediction model of sudden stop events of capital flows for both emerging countries and developed countries with the ability to estimate accurately future sudden stop scenarios globally. A sample of 103 countries was used, including 73 emerging countries and 30 developed countries, which has allowed the use of sample combinations that consider the regional heterogeneity of the warning indicators. To the sample under study, a method of decision trees has been applied, which has provided excellent prediction results given its ability to learn characteristics and create long-term dependencies from sequential data and time series. Our model has a great potential impact on the adequacy of macroeconomic policy against the risks derived from sudden stops of capital flows, providing tools that help to achieve financial stability at the global level.

## Introduction

**Fig 1:**
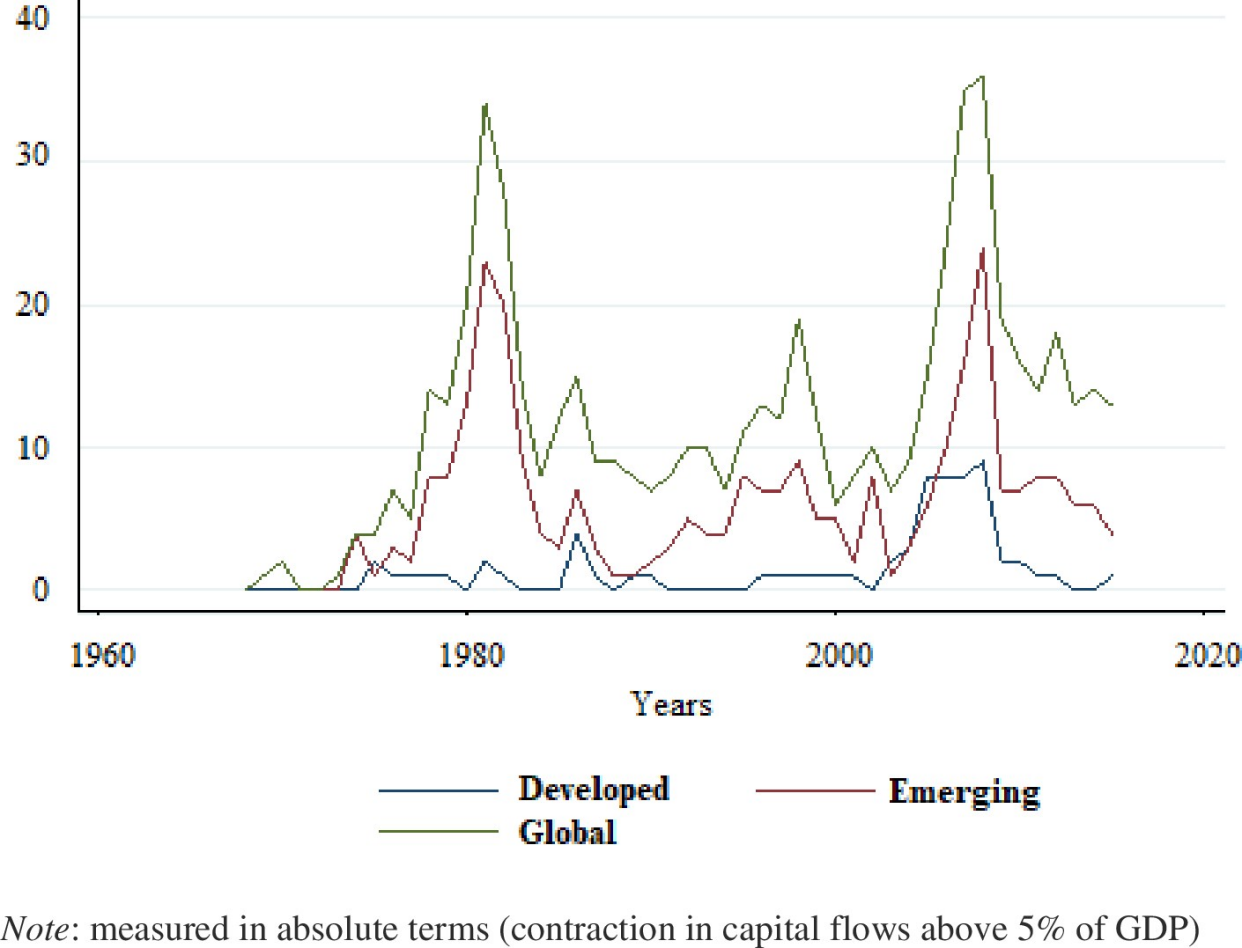
Number of sudden stops.

Sudden Stop (SS) is a sharp contraction of international capital flows. SS have significant negative effects on the global economy and as such have received special attention in the existing literature.[1] finds that SS lead to a drop in GDP growth of approximately 4%.[2] and [3] show that SS are accompanied by significant drops in production and employment. The financial crises caused by SS have a significant negative impact on output growth compared to currency crises. A currency crisis usually reduces output by about 2–3%, while a SS reduces output by an additional 6–8% in the year of the crisis [4]. Fig 1 shows the number of SS in absolute terms for developed countries, emerging economies and at the global level for the period 1960–2016. While emerging economies experienced more than 30 crises in 2009 alone, developed countries have not been immune to the phenomenon, having experienced more than ten crises in the last decade.

The recent literature contains a number of models for predicting SS [5–7]. These models have shown a preceding capital boom to be a good predictor of SS [8–10], together with increases in capital inflows accompanied by weak economic data and appreciated real exchange rates or high current account deficits [5,11,12]. However, the explanatory power of these models notwithstanding, there remain specific limitations in terms of their level of accuracy and their focus on groups of emerging countries. Hence, there is a need for further research on the problem, specifically in terms of new models that provide a better fit and extend the research problem beyond emerging countries to encompass developed countries [7,13].

To help make models for SS prediction more robust, this study has developed a new global model for predicting SS. The model has predictive capacity for all countries, with accuracy levels above 85%. The model was developed using a sample of 103 emerging and developed countries and uses artificial intelligence (AI) techniques applied to decision trees, a transparent decision-making mechanism. As such, our new model incorporates one of the requirements of the European Union recommendation on trustworthy AI, namely that technologies are robust, safe and transparent [14]. The guidelines recommend the incorporation of requirements to ensure trustworthy AI from the earliest design phase. These requirements are accountability, data governance, design for all, governance of AI autonomy (human oversight), non-discrimination, respect for human autonomy, respect for privacy, robustness, safety, transparency. The results obtained provide a significant improvement on the accuracy of previous models and contribute to literature on SS, providing experience for developed countries.

This study is structured as follows: Section 2 provides a literature review of empirical research on SS. Section 3 sets out the methodology used. Section 4 provides details of the data and the variables used in the research. Finally, section 5 analyses the results obtained. The article concludes by stating the conclusions of the study and their implications.

## Literature review

Existing literature on SS has three main lines of research. Firstly, there is analysis of the impacts of SS in different countries [15,16]. Secondly, there is the work that establishes a connection between increasing capital flows and SS [9,17,18]. Finally, there are studies that have developed models for predicting SS [5,6,7].

The effects of SS are accelerated slowdowns in economic activity and increased currency depreciation [19,15], significant drops in production and employment [2,3,16], falls in GDP [1,20] and financial crises [4].

Studies that have made the connection between increased capital flows and SS include [17], which analysed whether countries that limited international capital inflows were less likely to experience SS using a Probit model. The study found that high capital mobility is statistically significant and positive, with a small direct effect on the likelihood of a country experiencing a sharp drop in net capital inflows. In contrast, [18] found that countries that trade less with other countries have a greater propensity to SS and a currency collapse. In a study of 38 emerging economies covering the period 1990–2003, [5] found that a wave of capital inflows significantly increases the probability of SS. Moreover, the effect tends to be stronger if there is a large current account deficit or the real exchange rate has appreciated. [9] evaluated the effect of the “overreaction” in the stock market, showing that financial markets reacted excessively to new information or unexpected events. One of the main conclusions is that an upward overreaction subsequently causes a dramatic downward adjustment, in addition to an inexplicable high level of volatility in emerging countries. Subsequent research has also shown that after increased investment in emerging markets due to a significant rise in capital flows, numerous countries suffered a crisis with a large and unexpected reversal of capital flows [21].

The studies that have developed predictive models include [5]. After analysing 38 emerging markets in the period 1990–2003 using a Probit model, the study concluded that the relevant variables for predicting SS were Current Account to GDP and Real Exchange Rate. It obtained an accuracy of around 72%. [6] used a Logit model based on a sample of 43 countries for the period 1970–2009, separating the capital flows of countries into four components. They found that this separation helped better understand recent financial crises and improved prediction of SS compared to the standard two-way breakdown, with an accuracy of around 68%. The authors also concluded that the most significant variables for prediction were Current Account to GDP and Domestic Credit to GDP. Subsequently, [7] proposed a new model combining the two conventional approaches (signal extraction and logistic regression) to predict SS in emerging countries. The study identified the phenomenon of SS with Capital Flows to GDP Ratio, based on a sample of capital flows from 48 emerging countries for the period 1971–2014. The results showed that the model significantly improves predictive capacity and found that Current Account to GDP, External Debt to Exports Ratio, Terms of Trade, Real Exchange Rate and M2 –International Reserves Ratio were significant variables. Despite its results, the study found that the accuracy of the model (70%) could be significantly improved.

## Methodology

### Decision Tree and C5.0 algorithm

A decision tree (DT) is a graphical and analytic technique for classifying data in terms of different possible paths. Each node of the tree represents the different attributes of the data. The branches of the tree represent the possible paths to follow to predict the class of a new example. Finally, the terminal nodes or leaves establish the class of the test example in line with the branching in question. The notation used for describing the DTs is disjunctive normal form (DNF). Hence, if we have three attributes (A, B and C) each with two possible values x_i_ and ¬x_i_, where i = 1, 2, 3, there are 2^n^ possible combinations in DNF (n is the number of attributes). Each of the DNF combinations describes a part of the tree, giving the disjunctive forms expressed in Eq (1) for the tree.

(x2Λ¬x3)V(x2Λx3)V(¬x2Λx1)V(¬x2Λ¬x1) (1)

These disjunctions are descriptors of the tree that has been built. Thus it is possible to form 2^2n^ possible descriptions in DNF. Given that the order of DTs is extremely large, it is not possible to explore all the descriptors to identify the most adequate. Instead, heuristic search techniques are used to do this easily and quickly. The majority of the algorithms for constructing DTs are based on the Hill Climbing strategy. This is an AI technique used to find the maximums or minimums of the function via a local search. The algorithms begin with an empty tree, which is then segmented into sets of examples, in each case choosing the attribute that best discriminates between the classes until completing the tree. A heuristic function is used to find the best attribute and the choice is irrevocable, meaning it is important to ensure it is as close as possible to the optimal. The main advantage of using this type of strategy is the low computational cost.

There are various algorithms for building DTs. [22], which develops the so-called ID3 algorithm, is regarded as the seminal work in the field. The algorithm uses the notion of entropy to check the randomness of the distribution of a set of examples over the classes to which they belong. The C4.5 algorithm is an extension of ID3 and has the advantage of extracting hidden information from large datasets and providing classification rules with a high level of accuracy [23]. It builds a DT using partitions together with data. This construction is carried out using a “depth-first” strategy (all possible tests are used to divide the available data, selecting the one with the largest information gain). For each discrete attribute, a test with *n* possible results is considered In contrast, if the attributes are continuous, a single binary test is used for each of the values that the attribute can take. Every time a node is generated, the algorithm chooses the test as a function of the information gain provided, as expressed in (2).

$$Gain\;(S,A)=Entropy\;(S)-\sum_{i=1}^{n}\frac{\left|S_{i}\right|}{\left|S\right|}=Entropy\;(S)$$ (2)

where *S* is the set of cases, *A* is the attributes, *n* is the partition number of attribute *A*, and *S*_*i*_ is the number of cases in the i-th partition.

The entropy value is determined using Eq (3).

$$Entropy\;(S)=\sum_{i=1}^{n}-p_{i}*{log}_{2}*p_{i}$$ (3)

where *n* is the number of partitions of S and *p*_*i*_ is the proportion of *S*.

The C5.0 algorithm used in this study is a new-generation machine learning algorithm (MLA) based on DTs [24]. This means that DTs are built from the list of possible attributes and the set of training cases. The DTs can then be used to classify the remaining sets of test cases. The C5.0 algorithm offers a number of significant advantages over C4.5, since the rules generated are more accurate and it takes less time to generate them [25].

### Sensitivity analysis

Despite the significant explanatory capacity of DTs, when a large number of variables are used, it is also necessary to quantify their impact. This is done via the sensitivity analysis. This analysis aims to determine the relative importance of the independent variables in relation to the dependent variable [26]. It seeks to reduce the models to the most important variables and ignore or eliminate the least important. One variable is considered more important than another if it increases the variance, compared to the set of variables of the model. The Sobol method [27] is used to decompose the variance of the total output *V*(*Y*) provided by the set of equations expressed in (4).

$$V(Y)=\sum_{i}V_{i}+\sum_{i}\sum_{j>1}V_{ij}+\cdots +V_{12\ldots k}$$ (4)

Where $V_{i}=V(\frac{Y}{X_{i}}(E)$ and $V_{ij}=V(E(\frac{Y}{X_{i}X_{j}})-V_{i}$

For its part, the sensitivity indexes are determined by $Si=\frac{Vi}{V}$ and *Sij* = *Vij*/*V*, where *Sij* indicates the effect of interaction between two factors. The Sobol decomposition allows the estimation of a total sensitivity index *STi*, which measures the sum of all the sensitivity effects involved in the independent variables.

The DT methods used in this study are an appropriate measure of the sensitivity of the variables and are shown are shown in S1 Table.

## Sample, data and variables

### Sample and data

The chosen sample period is 1960–2016 for each of the three SS definitions specified above. The annual capital flow data has been used to identify SS events in 103 countries (73 emerging countries and 30 developed countries), permitting the construction of nine SS prediction models. Data from the IMF International Financial Statistics (IFS) and the World Bank has been used to classify countries and obtain information on the independent variables. Sudden stops events by country (global, emerging and developed) are shown in S1–S4 Figs, respectively. Sudden stops events by year (global, emerging and developed) are exhibited in S5–S7 Figs, respectively.

The sample data set has been divided into three groups mutually exclusive, one for training (70% of the data), another for validation (10% of the data) and a third group for testing (20% of the data). As is well known, the validation data is used to evaluate the decision tree during training, and to detect an over-training of it. If the error for the validation grows during a certain number of training times, the training is stopped. For its part, the testing data is used to evaluate the built model and make predictions. The percentage of correctly classified cases (accuracy) and the root of the mean square error have been used for the evaluation. Furthermore, for the treatment of each of the three groups, the 10-fold cross validation procedure has been applied with 500 iterations [28,29].

## Variables

The dependent variable used in this study is sudden stops of capital flows and it is a dummy variable, *Sj*,*t*, that takes a value of one for the occurrence of the SS events and zero otherwise for country *j* (*j* = 1, *J*) and at time t, that is expressed in (5).

$$S_{j,t}\equiv \left\{ \begin{array}{l} 1,\;crisis\\ 0,\;otherwise \end{array}\right.$$ (5)

It refers to the three definitions in [7,8,9] for Capital Flows to GDP ratio: SS1 (absolute and relative threshold), SS2 (absolute threshold), SS3 (relative threshold). These definitions correspond to the situations expressed in (6), (7) and (8), respectively.

$$S_{j,t}^{1}=\left\{ \begin{array}{c} 1,\;if\;\Delta {CF}_{j,t}<-5\%,\;\Delta {CF}_{j,t}<\bar{\Delta {CF}_{j,t}}-\sigma_{\Delta CFj}\\ 0,\;otherwise \end{array}\right.$$ (6)

$$S_{j,t}^{2}=\left\{ \begin{array}{c} 1,\;if\;\Delta {CF}_{j,t}<-5\%\\ 0,\;otherwise \end{array}\right.$$ (7)

$$S_{j,t}^{3}=\left\{ \begin{array}{c} 1,\;if\;\Delta {CF}_{j,t}<\bar{\Delta {CF}_{j,t}}-\sigma_{\Delta CFj}\\ 0,\;otherwise \end{array}\right.$$ (8)

where Δ*CF*_*j*,*t*_ denotes Capital Flows to GDP Ratio, $\bar{{\Delta CF}_{j,t}}$ indicates the historic average and $\sigma_{\Delta {CF}_{j}}$ the standard deviation.

**Fig 2:**
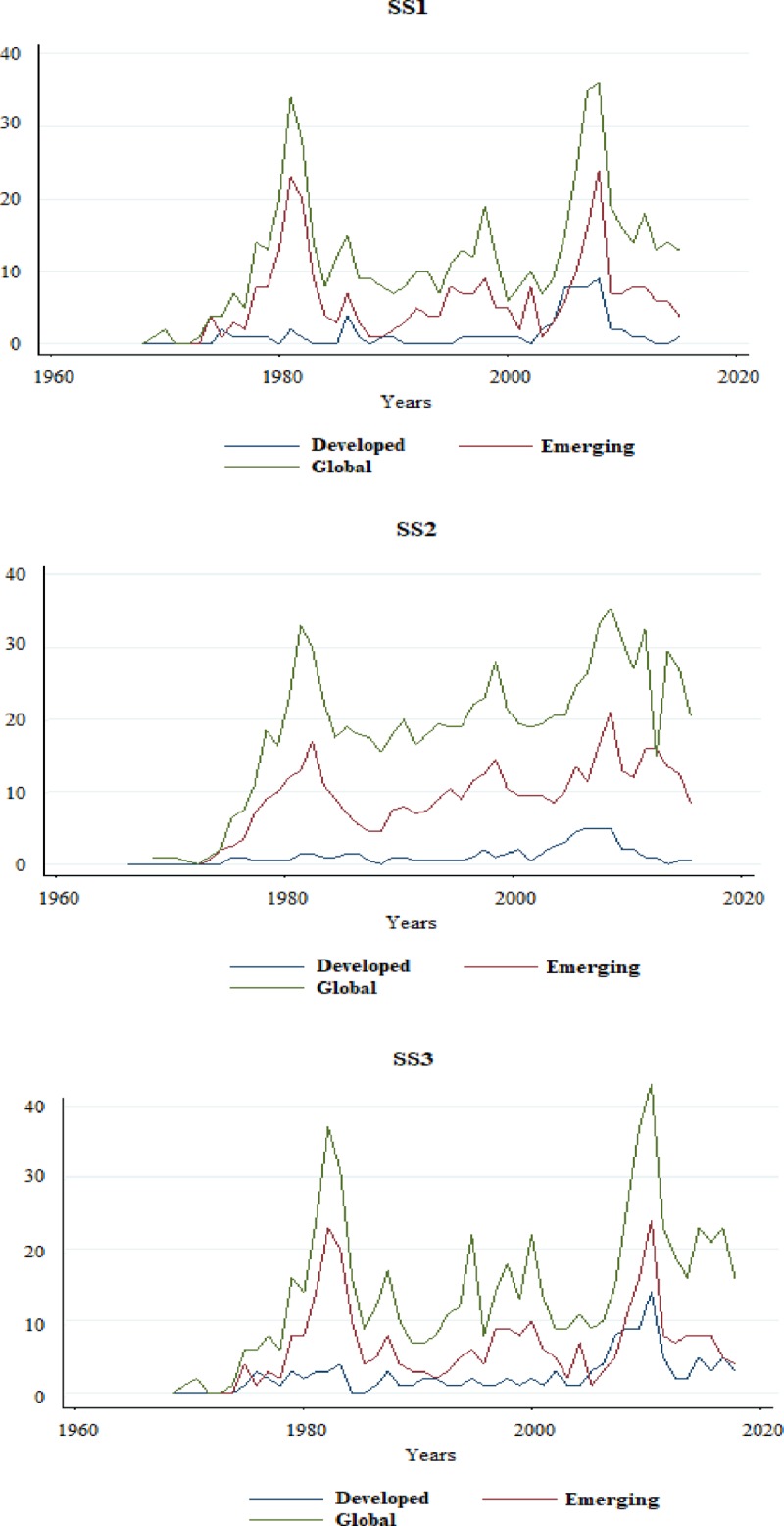
Dependent variable distribution.

Fig 2 shows the distribution of each of the three definitions (SS1, SS2, SS3) for the sample period used.

We used 36 independent variables as possible predictors of SS (Table 1). These are standard variables used in the existing literature [7,9,30,31], and they are classified according to their attributes (macroeconomic, financial, external, global and cross-country). The predictors have been built in period (t-1) regarding the measure of SS, that are in period t.

**Table 1:** Independent variables.

Attribute	Variables	Abbreviation	Expectedsign
Macroeconomic	Real GDP Growth (% annual)	RGDP	-
	Domestic Real Interest Rate (%)	DRINT	+
	Central Government Debt to GDP (% of GDP)	GDEBT	+
	Inflation (% annual)	INFLA	+
Financial	M2 Growth (% annual)	M2	+
	Depth of the Financial System (0 = low, 8 = high)	FDEPTH	-
	Return of Stock Market Index (% annual)	STOCK	-
	Domestic Credit to GDP (% of GDP)	CREDIT	+
External	Current Account to GDP (% of GDP)	CA	-
	External Debt to Exports Ratio (% of GNI)	EXDEBT	+
	Terms of Trade Adjustment (constant local currency)	TOT	-
	Real Exchange Rate (2010 = 100)	RER	+
	M2-International Reserves Ratio	FRES	+
	Foreign Direct Investment (% of GDP)	FDI	-
Global	GDP Growth of G7 Countries (% annual)	WGDP	-
	Foreign Interest Rate (% annual)	FINT	+
	VIX	VIX	+
	M2-Growth of World (% annual)	WM2	-
Cross-country	Exchange Rate Regime (1-floating, 2-fixed, 3-intermediate)	EXREG	-
	Openness (Imports +Exports/GDP)	OPEN	+
	GDP (per capita)	GDPCAP	-
	Capital Control (The Chinn-Ito Index)	CAPCON	-
	Geographic Proximity (dummy variable)	GEOPROX	+

## Results

### Descriptive statistics

**Fig 3:**
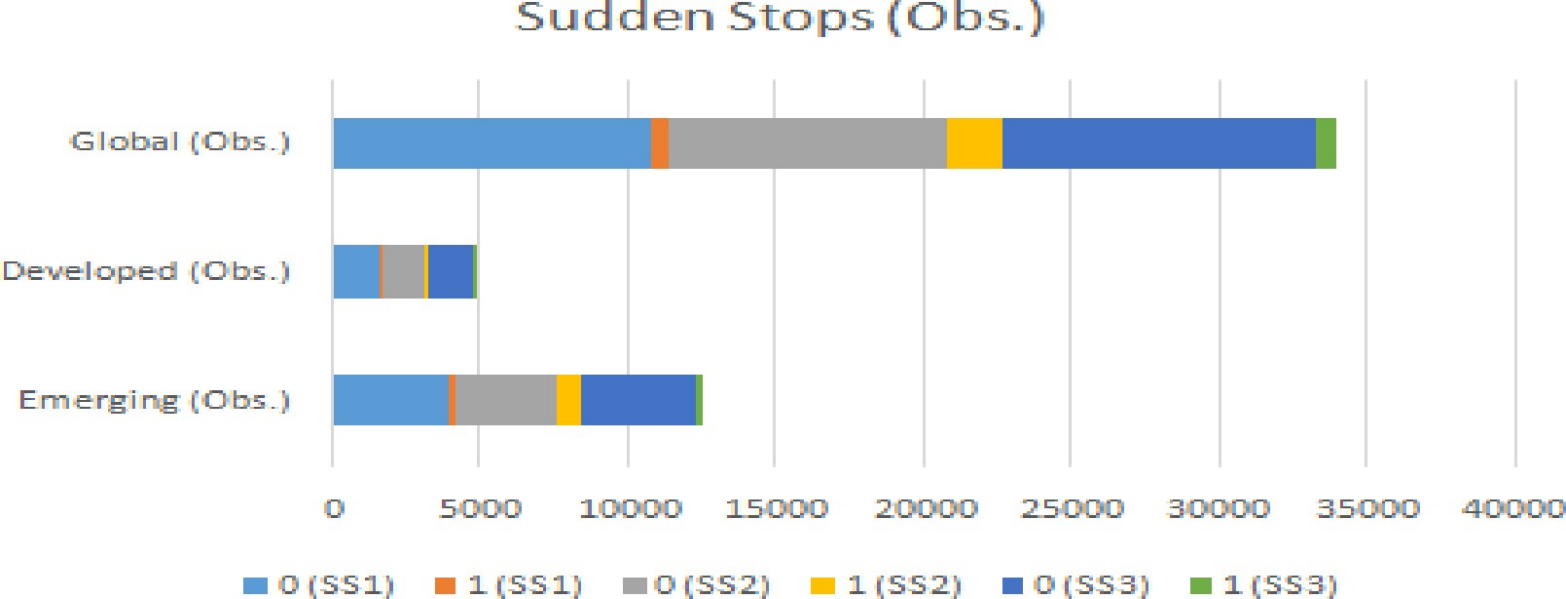
The transition matrices for SS (observations).

**Fig 4:**
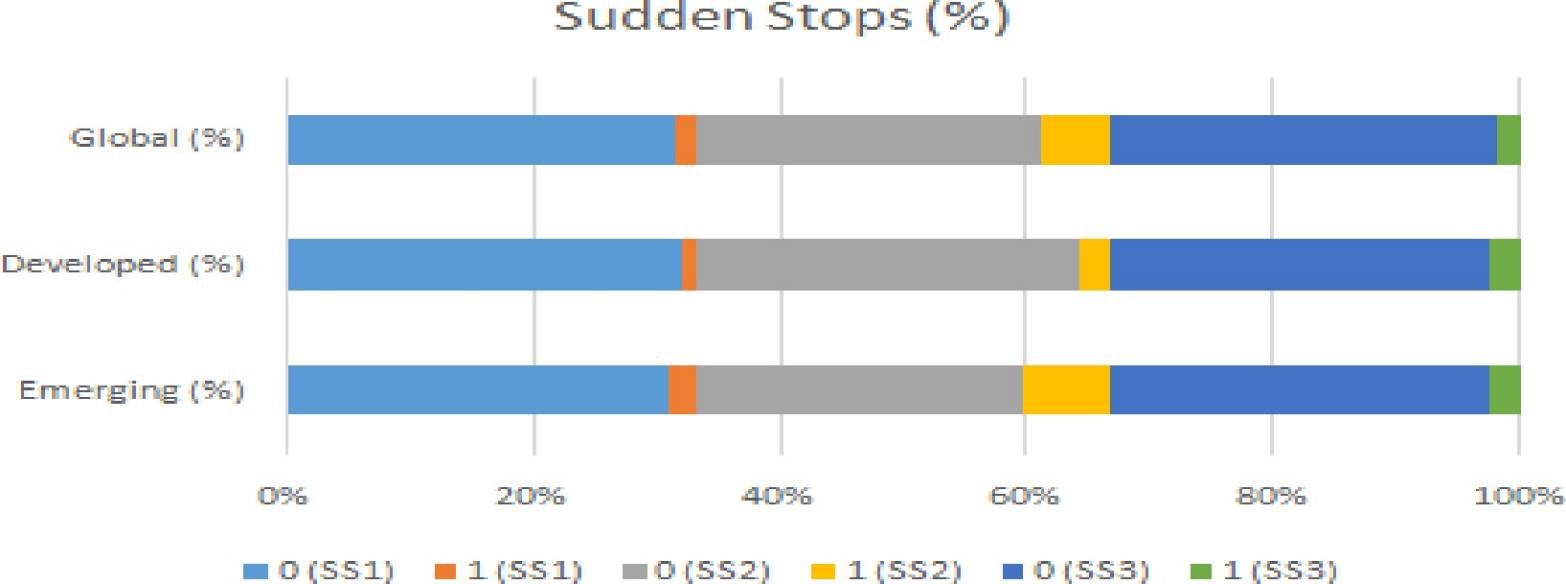
The transition matrices for SS (%).

The SS transition matrices in Figs 3 and 4 show the number of SS events in the three scenarios (SS1, SS2 and SS3) for each country category. In scenario SS2, emerging countries have experienced more SS events. Developed countries have experienced more SS events in scenario SS3. Comparing the sample of countries, the proportion of an SS event occurring in the three situations is in the range 18.48–3.56%.

Tables 2, 3 and 4 provide a statistical summary of the independent variables for emerging countries, developed countries and the overall sample (global). The average values for emerging countries are generally higher than those for developed countries. For example, the Real GDP Growth of emerging countries is 6.258%, compared to 3.131% for developed countries. This suggests that the economies of emerging countries are experiencing full economic development and, as such, grow faster than in the developed world. Similarly, the average External Debt to Export Ratio shows that emerging countries have a limited capacity to fund their external debt with exports. Furthermore, among the cases of negative values, the average value of the variable Capital Control in emerging countries is noteworthy, reflecting the fact that the quantity of products exported often fails to cover the quantity of products imported, in monetary terms.

**Table 2:** Emerging summary statistics.

Variables	Obs.	Mean	S.D.	Min.	Max.
Real GDP Growth	6,280	6.258	6.089	-27.270	19.300
Domestic Real Interest Rate	6,280	11.903	17.061	-7.977	77.617
Central Government Debt to GDP	6,280	57.710	4.121	50.298	67.484
Inflation	6,280	79.528	338.757	-3.793	2700.442
M2 Growth	6,280	110.447	415.903	3.212	3280.653
Depth Financial System	6,280	6.667	0.500	6.000	7.000
Return Stock Market Index	6,280	14.991	44.911	-64.137	125.110
Domestic Credit to GDP	6,280	50.229	35.436	7.708	153.341
Current Account to GDP	6,280	-0.223	2.837	-8.878	9.943
External Debt to Export Ratio	6,280	20.698	10.114	2.984	52.938
Terms of Trade Adjustment	6,280	-26803241.229	269371167.061	-1506211099.926	805997690.248
Real Exchange Rate Index	6,280	98.448	42.100	55.400	267.203
M2 International Reserves Ratio	6,280	8.814	6.251	1.932	54.280
GDP Growth G7 Countries	6,280			0.000	0.000
Foreign Interest Rate	6,280	4.245	2.637	0.561	9.305
VIX	6,280	15.685	1.370	14.176	17.520
M2 Growth of World	6,280	6.920	2.732	1.011	12.769
Exchange Rate Regime	6,280	1.833	0.842	1.000	3.000
Openness	6,280	38.625	7.382	3.057	86.214
GDP	6,280	1850.411	2812.155	70.909	13039.122
Capital control	6,280	-1.252	0.562	-1.895	0.393

**Table 3:** Developed summary statistics.

Variables	Obs	Mean	S.D.	Min.	Max.
Real GDP Growth	4,520	3.131	2.034	-3.202	7.167
Domestic Real Interest Rate	4,520	4.893	3.243	-4.267	12.232
Central Government Debt to GDP	4,520	31.303	23.797	3.673	78.103
Inflation	4,520	4.245	3.400	-2.294	16.469
M2 Growth	4,520	11.252	13.204	-25.551	125.031
Depth Financial System	4,520	6.667	1.323	5.000	8.000
Return Stock Market Index	4,520	7.786	24.912	-65.573	72.374
Domestic Credit to GDP	4,520	58.651	39.708	9.298	188.754
Current Account to GDP	4,520	-2.117	2.487	-7.522	4.486
External Debt to Export Ratio	4,520	73.318	34.594	3.892	119.416
Terms of Trade Adjustment	4,520	-16951022.401	26019.551	-90320976.649	44285992.997
Real Exchange Rate Index	4,520	95.493	12.396	66.824	121.048
M2 International Reserves Ratio	4,520	16.479	10.840	3.764	59.717
GDP Growth G7 Countries	4,520	3.231	2.198	-3.202	7.117
Foreign Interest Rate	4,520	3.857	2.103	0.417	8.531
VIX	4,520	13.825	1.121	14.097	16.547
M2 Growth of World	4,520	5.841	2.165	0.911	11.623
Exchange Rate Regime	4,520	1.255	0.580	1.000	3.000
Openness	4,520	54.907	6.526	8.269	159.145
GDP	4,520	19396.536	16358.760	1273.692	67652.683
Capital control	4,520	1.697	0.899	-0.126	2.389

**Table 4:** Global summary statistics.

Variables	Obs	Mean	S.D.	Min.	Max.
Real GDP Growth	8,490	4.695	4.062	-41.800	88.958
Domestic Real Interest Rate	8,490	8.398	10.152	-97.812	789.799
Central Government Debt to GDP	8,490	44.507	13.959	-4.906	11438.588
Inflation	8,490	41.886	171.079	-98.704	26762.018
M2 Growth	8,490	60.849	214.553	-99.875	108613.283
Depth Financial System	8,490	6.667	0.911	0.000	8.000
Return Stock Market Index	8,490	11.389	34.912	-84.230	912.281
Domestic Credit to GDP	8,490	54.440	37.572	0.000	312.154
Current Account to GDP	8,490	-1.170	2.662	-240.521	291.318
External Debt to Export Ratio	8,490	47.008	22.354	0.239	1380.766
Terms of Trade Adjustment	8,490	-21877131.815	14769.535	39147372539.354	25959303099.883
Real Exchange Rate Index	8,490	96.970	27.248	18.715	4162211.699
M2 International Reserves Ratio	8,490	12.647	8.546	-37.292	3986.518
GDP Growth G7 Countries	8,490	1.616	1.099	-5.619	12.882
Foreign Interest Rate	8,490	4.105	2.537	0.432	9.126
VIX	8,490	15.003	1.169	13.985	17.035
M2 Growth of World	8,490	6.756	2.564	0.923	12.156
Exchange Rate Regime	8,490	1.544	0.711	0.000	3.000
Openness	8,490	46.852	7.314	3.057	159.145
GDP	8,490	10623.473	9585.457	35.368	116612.884
Capital control	8,490	0.223	0.730	-1.895	2014.000

### Correlations

Tables 5, 6 and 7 provide the correlations between variables (dependent and independent) for emerging countries, developed countries and the overall sample (global). Correlations varied within a range between -0.312 and 0.322 in emerging countries, -0.328 and 0.332 in developed countries, and -0.228 and 0.262 in the global sample. According to the results obtained we can comment for example that SS3 has a high correlation with EXDEBT, CREDIT and GDEBT in emerging countries, developed countries and global sample, respectively.

**Table 5:** Correlations matrix (Emerging).

	RGDP	DRINT	GDEBT	INFLA	M2	FDEPTH	STOCK	CREDIT	CA	EXDEBT	TOT	RER	FRES	FDI	WGDP	FINT	VIX	WM2	EXREG	OPEN	GDPCAP	CAPCON	GEOPROX	SS1	SS2	SS3
RGDP	1	0.153	0.041	0.217	0.189	0.102	0.057	0.064	0.291	0.227	0.023	0.211	0.256	0.018	0.061	0.043	0.157	0.180	0.320	0.262	0.182	0.080	0.185	-0.087	-0.111	-0.108
DRINT		1	0.193	0.262	0.008	0.239	0.130	0.242	0.216	0.018	0.070	0.170	0.276	0.034	0.094	0.134	0.200	0.238	0.246	0.304	0.090	0.124	0.104	0.157	0.103	0.175
GDEBT			1	0.138	0.237	0.026	0.202	0.173	0.219	0.183	0.104	0.142	0.003	0.106	0.280	0.166	0.272	0.167	0.037	0.185	0.199	0.006	0.018	0.011	0.207	0.052
INFLA				1	0.239	0.191	0.275	0.073	0.052	0.055	0.162	0.169	0.275	0.064	0.106	0.290	0.002	0.037	-0.045	0.064	0.297	0.212	0.208	0.326	0.101	0.223
M2					1	0.042	0.281	0.007	0.137	0.214	0.140	0.174	0.169	0.094	0.007	0.270	0.058	0.025	0.182	0.320	0.006	0.292	0.300	0.196	0.253	0.255
FDEPTH						1	0.326	-0.175	0.097	0.184	-0.227	0.025	0.064	0.192	0.294	0.327	0.024	0.262	0.195	0.214	0.121	0.137	-0.147	-0.127	-0.244	-0.218
STOCK							1	0.200	0.120	0.022	0.314	0.241	0.163	0.174	0.081	0.197	0.301	0.039	0.077	0.319	-0.004	0.281	0.315	-0.050	-0.224	-0.172
CREDIT								1	0.215	0.189	0.151	0.064	0.123	0.191	0.236	0.274	0.108	-0.230	0.102	0.254	0.156	0.333	0.201	0.273	0.275	0.006
CA									1	0.249	0.051	0.282	0.098	0.112	0.042	-0.312	0.322	0.265	0.104	0.193	0.083	0.124	0.058	0.124	0.172	0.177
EXDEBT										1	0.066	0.184	0.277	0.092	-0.218	0.269	-0.011	0.233	0.148	0.029	0.249	0.115	0.196	0.144	0.289	0.293
TOT											1	0.288	0.037	0.017	0.125	0.021	0.211	0.270	0.136	0.037	0.050	0.272	0.271	-0.297	-0.047	-0.210
RER												1	0.093	0.119	0.142	0.294	0.277	0.192	0.285	0.151	0.227	0.144	0.297	0.063	0.147	0.034
FRES													1	0.034	0.097	0.048	0.314	-0.215	0.291	0.139	0.278	0.190	0.130	0.076	0.194	0.220
FDI														1	0.181	0.051	0.318	0.315	-0.206	0.182	0.167	0.135	0.054	-0.072	-0.117	-0.248
WGDP															1	0.104	0.069	0.282	0.320	0.223	0.234	0.254	0.186	-0.242	-0.227	-0.065
FINT																1	0.263	0.108	0.061	0.051	0.073	0.168	0.022	0.374	0.144	0.060
VIX																	1	0.208	-0.165	0.062	0.028	0.059	0.183	0.185	0.302	0.288
WM2																		1	0.036	0.221	0.222	0.143	-0.253	-0.167	-0.100	-0.292
EXREG																			1	0.143	0.089	0.118	0.031	-0.260	-0.216	-0.267
OPEN																				1	0.100	0.125	0.084	0.293	0.041	0.222
GDPCAP																					1	0.246	0.135	-0.285	-0.000	-0.288
CAPCON																						1	-0.007	-0.229	-0.117	-0.248
GEOPROX																							1	0.038	0.023	0.120
SS1																								1	0.135	0.227
SS2																									1	0.035
SS3																										1

**Table 6:** Correlations matrix (Developed).

	RGDP	DRINT	GDEBT	INFLA	M2	FDEPTH	STOCK	CREDIT	CA	EXDEBT	TOT	RER	FRES	FDI	WGDP	FINT	VIX	WM2	EXREG	OPEN	GDPCAP	CAPCON	GEOPROX	SS1	SS2	SS3
RGDP	1	0.235	0.160	0.288	0.230	0.226	0.186	0.047	0.062	0.306	0.101	0.228	0.158	0.191	0.270	0.170	0.229	0.033	0.329	0.322	0.216	0.006	0.241	-0.107	-0.297	-0.328
DRINT		1	0.128	0.200	0.332	-0.136	0.180	-0.103	0.154	0.189	0.136	0.228	0.234	0.224	0.265	0.204	0.250	0.132	0.251	0.068	0.109	0.063	0.111	0.334	0.163	0.261
GDEBT			1	0.177	0.123	0.142	0.299	0.135	0.255	0.158	0.031	0.168	0.221	0.162	0.154	0.093	0.236	0.006	0.013	0.300	0.147	0.186	0.326	0.206	0.259	0.249
INFLA				1	-0.006	0.326	-0.108	0.154	0.324	0.321	0.040	0.236	0.284	0.032	0.172	0.195	0.048	0.146	0.261	0.076	0.090	0.331	0.114	0.246	0.037	0.024
M2					1	0.197	0.185	0.187	0.057	0.303	0.155	0.129	0.211	0.041	0.329	0.024	0.176	0.029	0.022	0.138	0.159	0.011	0.061	0.207	0.192	0.282
FDEPTH						1	0.086	0.090	0.085	0.066	0.294	0.081	0.094	0.066	0.248	0.147	0.083	0.037	0.223	0.172	0.164	0.110	0.163	-0.019	-0.034	-0.130
STOCK							1	0.085	0.134	0.241	0.332	0.105	0.051	0.151	0.284	0.254	0.232	0.218	0.060	0.170	0.157	0.082	0.199	-0.210	-0.301	-0.328
CREDIT								1	0.220	0.287	0.296	0.242	0.152	0.163	0.273	0.107	0.150	0.295	0.056	0.111	0.275	0.199	0.328	0.195	0.287	0.232
CA									1	0.193	0.039	0.051	0.308	0.124	0.239	0.011	0.292	0.019	0.078	0.195	0.158	0.325	0.132	0.037	0.263	0.106
EXDEBT										1	0.062	0.282	0.023	0.088	0.021	0.243	0.324	0.288	0.122	0.064	0.001	0.129	0.039	0.269	0.005	0.064
TOT											1	0.142	0.142	0.104	0.242	0.039	0.267	0.184	0.175	0.218	0.321	0.066	0.027	-0.196	-0.118	-0.034
RER												1	0.047	0.143	0.313	0.103	0.060	0.189	0.266	0.049	0.329	0.188	0.036	0.082	0.117	0.118
FRES													1	0.159	0.075	0.059	0.081	0.192	0.280	0.201	0.298	0.288	0.185	0.274	0.130	0.139
FDI														1	0.114	-0.163	0.014	-0.117	0.041	0.113	0.041	0.205	0.142	-0.026	-0.132	-0.058
WGDP															1	0.293	0.291	0.287	0.318	0.033	0.143	0.273	0.291	-0.303	-0.142	-0.112
FINT																1	0.228	0.173	0.328	0.268	0.299	0.251	0.072	0.229	0.049	0.136
VIX																	1	0.213	0.187	0.314	0.301	0.291	0.072	0.152	0.303	0.330
WM2																		1	0.163	0.129	0.146	0.149	0.146	-0.251	-0.051	-0.141
EXREG																			1	0.098	0.218	0.164	0.174	-0.324	-0.282	-0.056
OPEN																				1	0.291	0.180	0.276	0.176	0.050	0.088
GDPCAP																					1	0.311	0.240	-0.279	-0.031	-0.127
CAPCON																						1	0.152	-0.261	-0.061	-0.121
GEOPROX																							1	0.047	0.113	0.176
SS1																								1	0.069	0.145
SS2																									1	0.093
SS3																										1

**Table 7:** Correlations matrix (Global).

	RGDP	DRINT	GDEBT	INFLA	M2	FDEPTH	STOCK	CREDIT	CA	EXDEBT	TOT	RER	FRES	FDI	WGDP	FINT	VIX	WM2	EXREG	OPEN	GDPCAP	CAPCON	GEOPROX	SS1	SS2	SS3
RGDP	1	0.235	0.160	0.288	0.230	0.226	0.186	0.047	0.062	0.306	0.101	0.228	0.158	0.192	0.270	0.170	0.229	0.033	0.329	0.322	0.216	0.006	0.241	-0.107	-0.097	-0.228
DRINT		1	0.128	0.200	0.332	0.136	0.180	0.103	0.154	0.189	0.136	0.228	0.234	0.062	0.265	0.204	0.250	0.132	0.251	0.068	0.109	0.063	0.111	0.034	0.193	0.061
GDEBT			1	0.177	0.123	0.142	0.299	0.135	0.255	0.158	0.031	0.168	0.221	0.142	0.154	0.093	0.236	0.006	0.013	0.300	0.147	0.186	0.326	0.106	0.159	0.241
INFLA				1	0.006	0.326	0.108	0.154	0.324	0.321	0.040	0.236	0.284	0.172	0.172	0.195	0.048	0.146	0.261	0.076	0.090	0.331	0.114	0.246	0.237	0.024
M2					1	0.197	0.185	0.187	0.057	0.303	0.155	0.129	0.211	0.205	0.329	0.024	0.176	0.029	0.022	0.138	0.159	0.011	0.061	0.007	0.182	0.262
FDEPTH						1	0.086	0.090	0.085	0.066	0.294	0.081	0.094	0.147	0.248	0.147	0.083	0.037	0.223	0.172	0.164	0.110	0.163	-0.019	-0.034	-0.230
STOCK							1	0.085	0.134	0.241	0.332	0.105	0.051	0.026	0.284	0.254	0.232	0.218	0.060	0.170	0.157	0.082	0.199	-0.010	-0.301	-0.128
CREDIT								1	0.220	0.287	0.296	0.242	0.152	0.163	0.273	0.107	0.150	0.295	0.056	0.111	0.275	0.199	0.328	0.195	0.087	0.182
CA									1	0.193	0.039	0.051	0.308	0.016	0.239	0.011	0.292	0.019	0.098	0.195	0.158	0.325	0.132	0.037	0.063	0.126
EXDEBT										1	0.062	0.282	0.023	0.048	0.021	0.243	0.324	0.288	0.107	0.064	0.001	0.129	0.039	0.169	0.005	0.164
TOT											1	0.142	0.142	0.192	0.242	0.039	0.267	0.184	0.175	0.218	0.321	0.066	0.027	-0.296	-0.218	-0.034
RER												1	0.047	0.157	0.313	0.103	0.060	0.189	0.266	0.049	0.329	0.188	0.036	0.082	0.117	0.018
FRES													1	0.092	0.075	0.059	0.081	0.192	0.280	0.201	0.298	0.288	0.185	0.074	0.130	0.239
FDI														1	-0.162	0.114	0.015	0.041	0.135	-0.076	0.315	0.044	0.062	-0.085	-0.104	-0.074
WGDP															1	0.293	0.291	0.287	0.318	0.033	0.143	0.273	0.291	-0.303	-0.132	-0.212
FINT																1	0.228	0.173	0.328	0.268	0.299	0.251	0.072	0.229	0.049	0.236
VIX																	1	0.213	0.187	0.314	0.301	0.291	0.072	0.052	0.203	0.030
WM2																		1	0.163	0.129	0.146	0.149	0.146	-0.151	-0.251	-0.161
EXREG																			1	0.098	0.218	0.164	0.174	-0.174	-0.282	-0.216
OPEN																				1	0.291	0.180	0.276	0.176	0.050	0.088
GDPCAP																					1	0.311	0.240	-0.079	-0.031	-0.127
CAPCON																						1	0.152	-0.161	-0.061	-0.021
GEOPROX																							1	0.047	0.123	0.146
SS1																								1	0.069	0.125
SS2																									1	0.093
SS3																										1

### Empirical results

**Fig 5:**
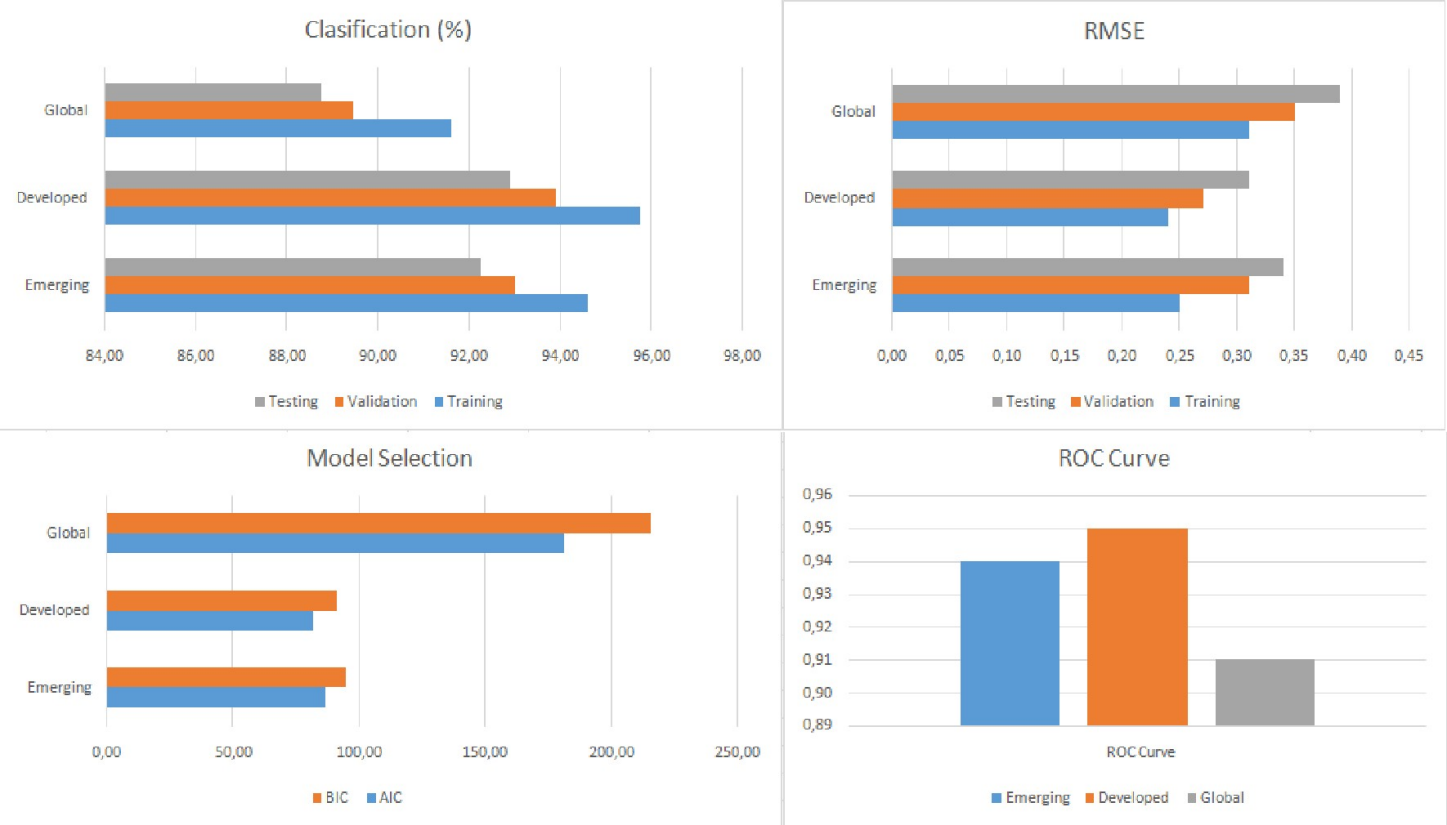
Results of accuracy evaluation for SS1.

**Fig 6:**
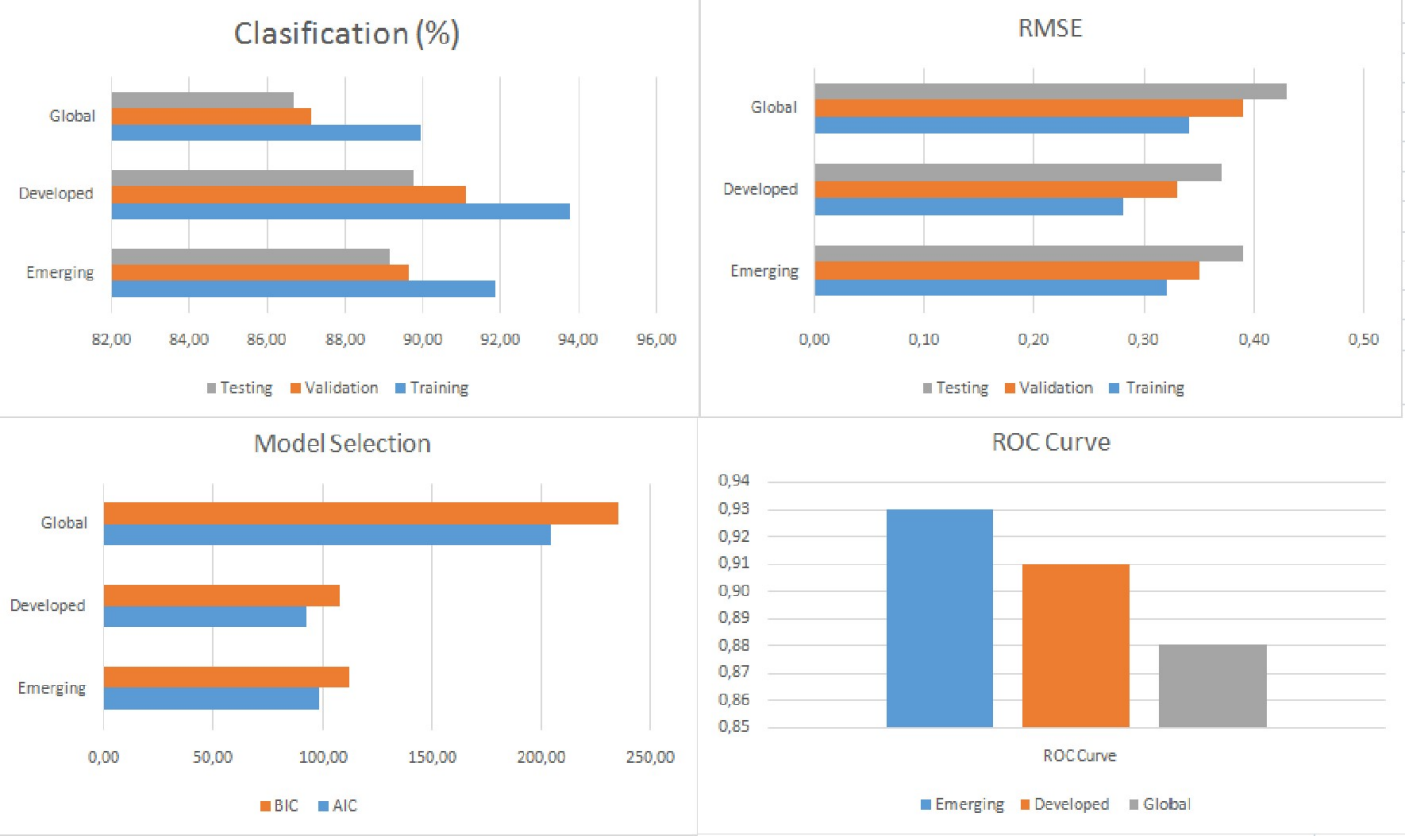
Results of accuracy evaluation for SS2.

**Fig 7:**
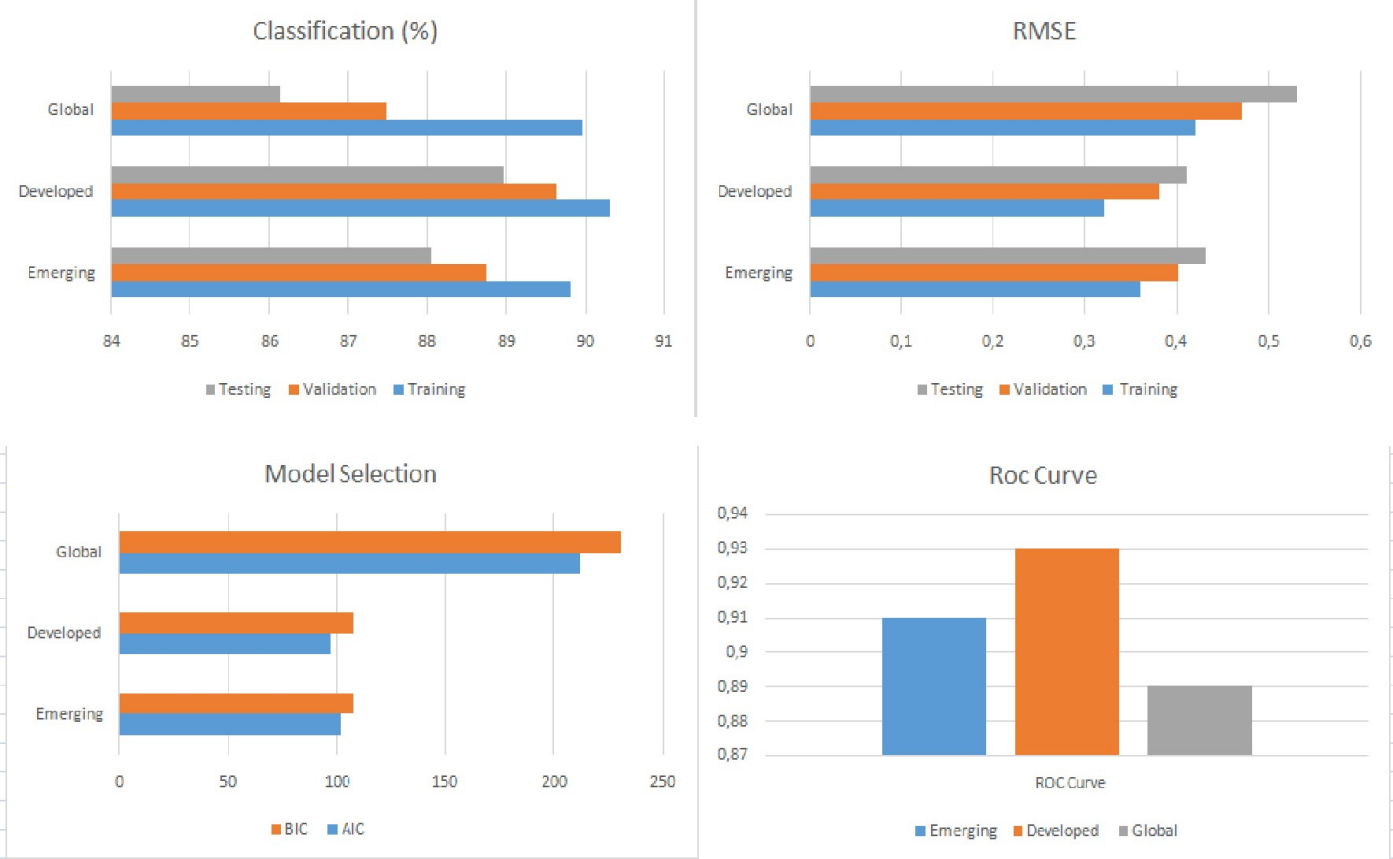
Results of accuracy evaluation for SS3.

Tables 8, 9 and 10 and Figs 5, 6 and 7 show the accuracy level, root mean square error (RMSE), the model selection criteria, the ROC curve value and the variables with the greatest sensitivity for each of the models produced. In all cases, accuracy is higher than 87.12% and both the RMSE levels and ROC values are adequate. The model with the highest accuracy value (93.89%) is for developed countries in SS1, followed by the model for emerging countries in SS1 (93.02%). As a whole, these results provide a much higher level of accuracy than previous research. The accuracy in the study by [7] is around 70% and the figure for the study by Janus and Riera-Crichton [6] is around 68%.

**Table 8:** Results of accuracy evaluation for SS1.

Sample	Classification (%)			RMSE			Model Selection		ROC Curve	Greater sensitivity variables
	Training	Validation	Testing	Training	Validation	Testing	AIC	BIC		
Emerging	94.58	93.02	92.25	0.25	0.31	0.34	86.18	94.85	0.94	INFLA, CREDIT, TOT, WM2, WGDP, FINT, VIX, EXREG
Developed	95.76	93.89	92.88	0.24	0.27	0.31	81.49	90.97	0.95	DRINT, INFLA, M2, STOCK, TOT, VIX, WM2, EXREG
Global	91.61	89.45	88.74	0.31	0.35	0.39	181.22	215.37	0.91	RGDP, GDEBT, M2, FDEPTH, CREDIT, TOT, WGDP, FINT, EXREG

RSME: Root of the Mean Square Error; AIC: Akaike Information Criteria; BIC: Bayesian Information Criteria.

**Table 9:** Results of accuracy evaluation for SS2.

Sample	Classification (%)			RMSE			Model Selection		ROC Curve	Greater sensitivity variables
	Training	Validation	Testing	Training	Validation	Testing	AIC	BIC		
Emerging	91.84	89.62	89.14	0.32	0.35	0.39	97.95	111.541	0.93	M2, STOCK, CA, EXDEBT, FRES, WGDP, VIX. EXREG
Developed	93.76	91.07	89.73	0.28	0.33	0.37	91.74	107.41	0.91	RGDP, DRINT, GDEBT, M2, STOCK, CREDIT, CA, VIX
Global	89.93	87.12	86.65	0.34	0.39	0.43	204.21	235.16	0.88	DRINT, GDEBT, M2, STOCK, CREDIT, CA, EXDEBT, FRES, WGDP, VIX

RSME: Root of the Mean Square Error; AIC: Akaike Information Criteria; BIC: Bayesian Information Criteria.

**Table 10:** Results of accuracy evaluation for SS3.

Sample	Classification (%)			RMSE			Model Selection		ROC Curve	Greater sensitivity variables
	Training	Validation	Testing	Training	Validation	Testing	AIC	BIC		
Emerging	89.83	88.76	88.06	0.36	0.40	0.43	101.77	108.16	0.91	RGDP, INFLA, M2, STOCK, CA, EXDEBT, FRES, FDI, EXREG
Developed	90.32	89.64	88.97	0.32	0.38	0.41	96.75	108.02	0.93	RGDP, DRINT, GDEBT, M2, STOCK, CREDIT, VIX
Global	89.97	87.74	86.13	0.42	0.47	0.53	212.11	230.98	0.89	RGDP, GDEBT, INFLA, FDEPTH, CREDIT, EXDEBT, VIX, WM2

RSME: Root of the Mean Square Error; AIC: Akaike Information Criteria; BIC: Bayesian Information Criteria.

**Fig 8:**
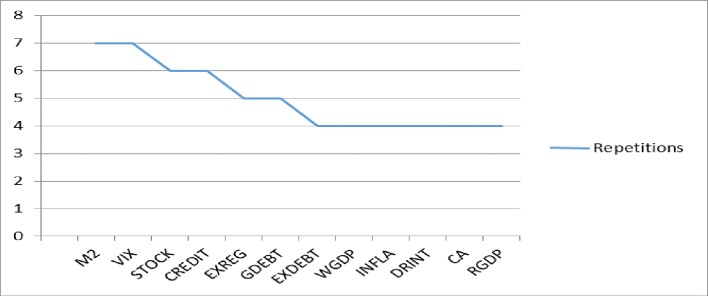
Number of repetitions of the greater sensitivity variables.

**Fig 9:**
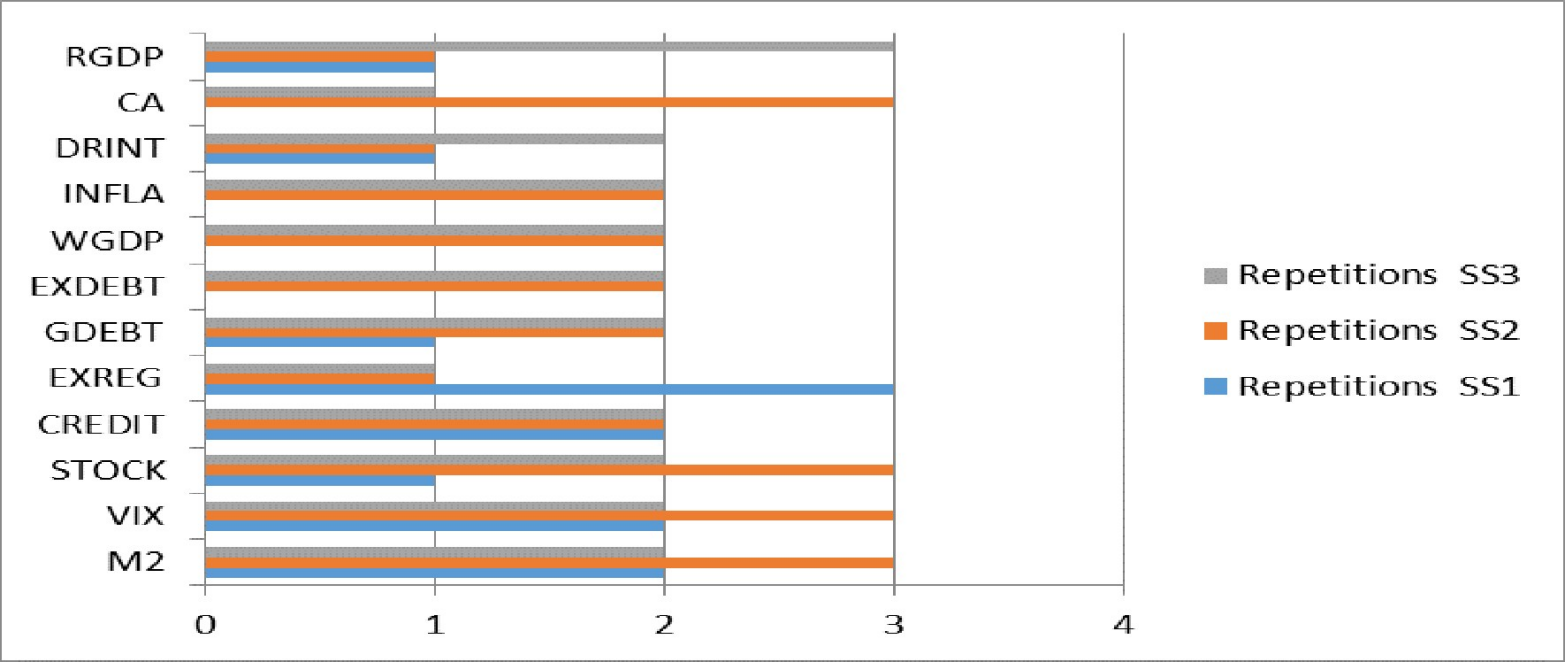
Number of repetitions of the greater sensitivity variables in SS1, SS2 and SS3.

Figs 8 and 9 provide additional information on the greater sensitivity variables (see also S1 Table). The variables M2 and VIX are significant in seven models (twice in SS1, three times in SS2 and twice in SS3). The variables STOCK and CREDIT, both from the subgroup of financial variables, are repeated in six models (STOCK once in SS1, three times in SS2 and twice in SS3; and CREDIT twice in SS1 and twice again in both SS2 and SS3). The variables EXREG and GDEBT appear in five models (EXREG three times in SS1 and once in both SS2 and SS3; and GDEBT once in SS1 and twice in SS2 and SS3). Other variables (DRINT, CA, RGDP, EXDEBT, WGDP and INFLA) are repeated in four models. The variables EXDEBT, WGDP and INFLA follow the same patterns, twice in SS2 and SS3. The variable DRINT appears once in SS1 and SS3, and twice in SS2. The variable CA appears three times in SS2 and once in SS3. Finally, the variable RGDP is significant once in SS1 and SS2 and twice in SS3.

The results show that for emerging countries, the significant variable that appears in the three SS scenarios is EXREG (subgroup of cross-country variables). Moreover, the variables INFLA, WDGP, STOCK, CA, EXDEBT, FRES and VIX are repeated twice. Compared to other previous research, the variables CA, EXDEBT, TOT, RER and FRES are also significant in the study by [7]. Similarly, CA and CREDIT were significant in the study by [6], and CA and RER in the study by [5]. This shows that our research has validated new significant variables in the macroeconomic, financial, global and cross-country subgroups (EXREG, INFLA, WDGP, STOCK and VIX) and thus identifies a new set of significant variables that differ from previous research.

The results for the three models for developing countries show that DRINT, M2, STOCK, VIX, RGDP, GDEBT and CREDIT are the variables with greatest sensitivity for predicting SS. Hence, developed countries must be alert to the behaviour of these variables, since high real interest rates, public debt to GDP, M2 growth, the level of domestic credit and the volatility index are all linked to a higher probability of an SS event. Likewise, higher GDP growth and the performance of the stock index are negatively related to the possibility of an SS event. Since there is no previous research on forecasting specifically for developed countries, the results of this research represent an innovative contribution to the literature on SS.

Finally, regarding the results of the global models, it can be deduced that the variables with greatest sensitivity for predicting SS in the three scenarios considered are GDEBT, M2, CREDIT, FDEPTH, RGDP, EXDEBT and VIX. There are no previous studies on predicting SS at the global level to compare with our results. However, our research provides new significant variables to be considered for predicting SS in any country. These variables are GDEBT, M2, CREDIT, FDEPTH, RGDP, EXDEBT and VIX, with M2 and VIX standing out, since these appear with greatest frequency in the nine models considered.

### Post-estimations

**Fig 10:**
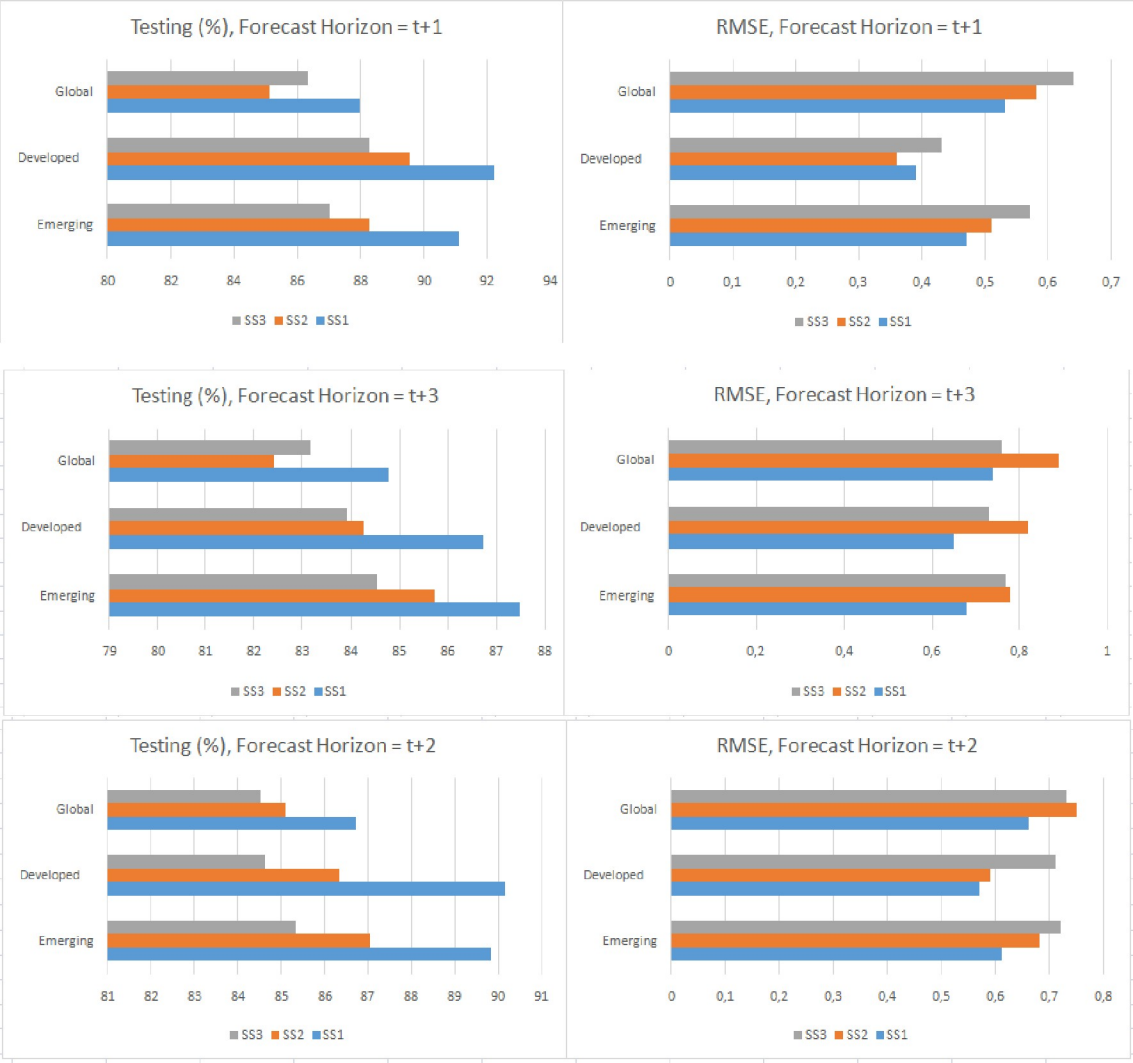
Multiple-step ahead forecasts and RMSE score.

Using multiple-step-ahead prediction, we have considered the iterative strategy, where models that are trained for the prediction of 1-step forward are developed [32]. At time t, a prediction is made for moment t+1, and this prediction is used to make the prediction for moment t+2 and so on. This means that the predicted data for t+1 are considered real data and are added to the end of the available data [33].Table 11 and Fig 10 show the accuracy and error results for t+1, t+2 and t+3 forecasting horizons. For t+1, the highest accuracy values for each model occur in the scenario SS1, with a range of 92.40–85.21%. The highest accuracy occurs in the models for developed countries. The highest level of accuracy for t+2 is also for developing countries in scenario SS1 (90.21%). In this case the accuracy range is 90.21–84.47%. The accuracy range for t+3 is 87.38–82.49% and the highest value also occurs in SS1 (emerging countries, with 87.38%). These results show a high level of accuracy for the three prediction horizons, together with the high stability of the models, since this accuracy only slightly decreases as the horizon increases (the t+1 accuracy of the emerging countries model for SS1 is 91.21%, dropping to 89.77% in t+2 and 87.38% in t+3).

**Table 11:** Multiple-step ahead forecasts and RMSE score.

Sample		Forecast horizon = t+1			Forecast horizon = t+2			Forecast horizon = t+3		
		SS1	SS2	SS3	SS1	SS2	SS3	SS1	SS2	SS3
Emerging	Classification (%)	91.21	88.18	87.19	89.77	87.12	85.27	87.38	85.79	84.46
	RMSE	0.46	0.51	0.57	0.61	0.68	0.73	0.68	0.78	0.79
Developed	Classification (%)	92.40	89.68	88.42	90.21	86.38	84.71	86.68	84.33	83.86
	RMSE	0.38	0.36	0.43	0.57	0.59	0.71	0.65	0.82	0.74
Global	Classification (%)	87.91	85.21	86.39	86.81	85.13	84.47	84.73	82.49	83.23
	RMSE	0.53	0.58	0.64	0.65	0.75	0.73	0.74	0.88	0.76

## Conclusions

This study has developed new models for predicting SS for emerging countries, developed countries and a global sample of countries. It has applied DTs as an innovative method not used in previous research in the field. Specifically, the goal has been to improve the predictive accuracy of previous studies using different methodologies and increase the sample size to all countries in the world. The results obtained in this research are significantly higher than those obtained in the existing literature, with an accuracy range of 87.12–93.89%. Our improvement in accuracy may also be due to greater coverage of years and countries of our sample with respect to other previous works, and this should also be considered for future work. It has also detected new significant variables in these SS prediction models, allowing a high level of stability in the models developed over forecasting horizons t+1, t+2 and t+3.

In contrast to previous research, this study has been able to expand predictions of SS events beyond emerging countries to the global level. The results have identified different significant variables for emerging countries and developed countries, as well as at the global level. This makes an essential contribution to the field of international finance. The conclusions are relevant to agents responsible for economic policy in any country in the world, since our study suggests new explanatory significant variables to allow political agents to predict SS phenomena. This research has also provided a new SS forecasting model developed using DTs, thus contributing to existing knowledge in the field of AI. This new model can be used as a reference for setting macroeconomic policy and improved decision-making.

In summary, this study provides a significant opportunity to contribute to the field of finance, since the results obtained have significant implications for the future decisions of political agents, making it possible to avoid SS events and the potential associated costs. It also helps these agents send warning signals to financial markets and avoid financial crises derived from the phenomenon of SS.

A limitation of this study is that there are possible cases of countries that have changed from a situation of emerging countries to developed countries in our sample period. In this work we have not taken it into account for the purpose of greater homogeneity, but could be future lines of research.

Opportunities for further research in this field include developing predictive models taking into account political factors that evaluate the possible influence of the management and effectiveness of economic policy on the phenomenon of SS.

## Supporting information

S1 Fig Sudden Stop events by country (global). These figures show the number of SS events according to three definitions (SS1, SS2 and SS3).
(DOCX)

S2 Fig Sudden Stop events by country (emerging). These figures show the number of SS events according to three definitions (SS1, SS2 and SS3).
(DOCX)

S3 Fig Sudden Stop events by country (emerging) (continued). (DOCX)

S4 Fig Sudden Stop events by country (developed). This figure shows the number of SS events according to three definitions (SS1, SS2 and SS3).
(DOCX)

S5 Fig Sudden Stop events by year (global). This figure shows the number of SS events according to three definitions (SS1, SS2 and SS3) by year.
(DOCX)

S6 Fig Sudden Stop events by year (emerging). This figure shows the number of SS events according to three definitions (SS1, SS2 and SS3) by year.
(DOCX)

S7 Fig Sudden Stop events by year (developed). This figure shows the number of SS events according to three definitions (SS1, SS2 and SS3) by year.
(DOCX)

S1 Table Variable importance values of ratios for Sudden Stop. This table shows the variable importance values of ratios for Sudden Stop after applying the sensitivity analysis.
(DOCX)

S1 File Sample. Data file with all the historical information used with the independent variables and the dependent binary variable.
(DOCX)
