# Supplementary material for: A global prediction model for sudden stops of capital flows using decision trees
Source: PLoS One. 2020 Feb 12;15(2):e0228387. doi: 10.1371/journal.pone.0228387 (PMC7015411; doi:10.1371/journal.pone.0228387)
Supplement: S3 Fig — (DOCX) [file pone.0228387.s003.docx]

**S3 Fig. Sudden Stop events by country (emerging) (continued).**
