# Supplementary material for: A global prediction model for sudden stops of capital flows using decision trees
Source: PLoS One. 2020 Feb 12;15(2):e0228387. doi: 10.1371/journal.pone.0228387 (PMC7015411; doi:10.1371/journal.pone.0228387)
Supplement: S4 Fig — This figure shows the number of SS events according to three definitions (SS1, SS2 and SS3). (DOCX) [file pone.0228387.s004.docx]

**S4 Fig. Sudden Stop events by country (developed).**

This figure shows the number of SS events according to three definitions (SS1, SS2 and SS3).
