# Supplementary material for: A global prediction model for sudden stops of capital flows using decision trees
Source: PLoS One. 2020 Feb 12;15(2):e0228387. doi: 10.1371/journal.pone.0228387 (PMC7015411; doi:10.1371/journal.pone.0228387)
Supplement: S1 Table — This table shows the variable importance values of ratios for Sudden Stop after applying the sensitivity analysis. (DOCX) [file pone.0228387.s008.docx]

**S1 Table. Variable importance values of ratios for Sudden Stop.**

This table shows the variable importance values of ratios for Sudden Stop after applying the sensitivity analysis.

| Variables | SS1 | | | SS2 | | | SS3 | | |
| --- | --- | --- | --- | --- | --- | --- | --- | --- | --- |
|  | Emerging | Developed | Global | Emerging | Developed | Global | Emerging | Developed | Global |
| RGDP | 0.0000 | 0.0000 | 1.0000 | 0.0000 | 1.0000 | 0.0000 | 0.0860 | 0.1710 | 0.0000 |
| DRINT | 0.0000 | 0.3060 | 0.0000 | 0.0000 | 0.0240 | 0.0420 | 0.0000 | 0.0750 | 0.0000 |
| GDEBT | 0.0000 | 0.0000 | 0.1570 | 0.0000 | 0.1830 | 0.1490 | 1.0000 | 0.2420 | 0.0000 |
| INFLA | 0.2630 | 0.2730 | 0.0000 | 0.0000 | 0.0000 | 0.0000 | 0.0730 | 0.0000 | 0.0000 |
| M2 | 0.0000 | 1.0000 | 0.2480 | 0.1520 | 0.2510 | 0.1730 | 0.1280 | 0.0340 | 0.0000 |
| FDEPTH | 0.0000 | 0.0000 | 0.0350 | 0.0000 | 0.0000 | 0.0000 | 0.0000 | 0.0000 | 0.0000 |
| STOCK | 0.0000 | 0.4520 | 0.0000 | 1.0000 | 0.2370 | 0.0480 | 0.2570 | 0.2710 | 0.0000 |
| CREDIT | 1.0000 | 0.0000 | 0.1720 | 0.0000 | 0.1810 | 1.0000 | 0.0000 | 1.0000 | 0.0000 |
| CA | 0.0000 | 0.0000 | 0.0000 | 0.0590 | 0.0720 | 0.1850 | 0.1620 | 0.0000 | 0.0000 |
| EXDEBT | 0.0000 | 0.0000 | 0.0000 | 0.2040 | 0.0000 | 0.0720 | 0.0550 | 0.0000 | 0.0000 |
| TOT | 0.3470 | 0.1820 | 0.2100 | 0.0000 | 0.0000 | 0.0000 | 0.0000 | 0.0000 | 0.0000 |
| RER | 0.0000 | 0.0000 | 0.0000 | 0.0000 | 0.0000 | 0.0000 | 0.0000 | 0.0000 | 0.0000 |
| FRES | 0.0000 | 0.0000 | 0.0000 | 0.0840 | 0.0000 | 0.2140 | 0.1520 | 0.0000 | 0.0000 |
| WGDP | 0.0930 | 0.0000 | 0.1860 | 0.1050 | 0.0000 | 0.0820 | 0.0000 | 0.0000 | 0.0000 |
| FINT | 0.0526 | 0.0000 | 0.0730 | 0.0000 | 0.0000 | 0.0000 | 0.0000 | 0.0000 | 0.0000 |
| VIX | 0.1490 | 0.2680 | 0.0000 | 0.0630 | 0.1340 | 0.1300 | 0.0000 | 0.1420 | 0.0000 |
| WM2 | 0.1720 | 0.0470 | 0.0000 | 0.0000 | 0.0000 | 0.0000 | 0.0000 | 0.0000 | 0.0000 |
| EXREG | 0.2040 | 0.0430 | 0.1790 | 0.1170 | 0.0000 | 0.0000 | 0.1430 | 0.0000 | 0.0000 |
| OPEN | 0.0000 | 0.0000 | 0.0000 | 0.0000 | 0.0000 | 0.0000 | 0.0000 | 0.0000 | 0.0000 |
| GDPCAP | 0.0000 | 0.0000 | 0.0000 | 0.0000 | 0.0000 | 0.0000 | 0.0000 | 0.0000 | 0.0000 |
| CAPCON | 0.0000 | 0.0000 | 0.0000 | 0.0000 | 0.0000 | 0.0000 | 0.0000 | 0.0000 | 0.0000 |
| GEOPROX | 0.0000 | 0.0000 | 0.0000 | 0.0000 | 0.0000 | 0.0000 | 0.0000 | 0.0000 | 0.0000 |
