# Supplementary material for: A global prediction model for sudden stops of capital flows using decision trees
Source: PLoS One. 2020 Feb 12;15(2):e0228387. doi: 10.1371/journal.pone.0228387 (PMC7015411; doi:10.1371/journal.pone.0228387)
Supplement: S1 File — Data file with all the historical information used with the independent variables and the dependent binary variable. (DOCX) [file pone.0228387.s009.docx]

**S1 File.** Sample. Data file with all the historical information used with the independent variables and the dependent binary variable. (XLSX)
